# Supplementary material for: Chromosome-Level Genome Assembly and Transcriptome Comparison Analysis of Cephalopholis sonnerati and Its Related Grouper Species
Source: Biology (Basel). 2022 Jul 13;11(7):1053. doi: 10.3390/biology11071053 (PMC9312885; doi:10.3390/biology11071053)
Supplement: Supplementary file 1 [file biology-11-01053-s001.zip › Supplement tables and figures.pdf]

Supplementary Materials

Chromosome-Level Genome Assembly and Transcriptome Comparison Analysis of *Cephalopholis sonnerati* and Its Related Grouper Species

Zhenzhen Xie, Dengdong Wang, Shoujia Jiang, Cheng Peng, Qing Wang, Chunren Huang, Shuisheng Li, Haoran Lin and Yong Zhang

Table S1. Sequencing data for the *C. sonnerati* genome assembly.

Sequencing librar-ies	Insert size (bp)	Polymerase reads (Gb)	Subreads (Gb)	Mean read length (bp)	Sequence coverage (×)
BGI reads	350	59.70	57.12	148.81	54.70
Pacbio reads	400,00	152.28	152.21	231,09	145.80
Hi-C reads	350	124.03	118.96	148.36	113.94
Total	40,700	336.02	328.29	23,406.17	314.44

Table S2. Statistics of *C. sonnerati* genome size, heterozygosity, and repeat ratio.

Sample	*C. sonnerati*
K-mer	17
K-mer number	50,816,125,757
K-mer depth	47
Genome size (Mbp)	1,015
Heterozygosity ratio (%)	0.84
Repeat (%)	42.99

Table S3. Statistics of the subreads in *C. sonnerati* genome.

Subreads base (G)	Subreads number	Average subreads length (bp)	N50 (bp)
152.21	65,868,118	23,109	32,006

Table S4. *De novo* assembly of *C. sonnerati* genome using chromatin interaction mapping. *Number of scaffolds: 24 (100% of all contigs in chromosome clusters, 98.04% of all contigs).

Scaffold number	Number of contigs	Length (bp)
1	23	50,841,404
2	23	50,311,555
3	34	48,754,761
4	40	47,023,485
5	23	46,734,807
6	33	45,893,910
7	30	45,164,714
8	32	45,162,194
9	39	44,645,089
10	32	44,640,547
11	35	44,465,143
12	26	43,984,600
13	29	42,837,645
14	30	42,700,755

15	50	42,208,677
16	29	42,135,299
17	27	41,894,452
18	28	40,877,315
19	33	39,230,152
20	34	39,215,218
21	32	39,215,218
22	38	36,779,769
23	42	35,404,564
24	25	23,261,553
Total	767	1,022,871,484
N50		44,482,143

Table S5. Statistics of the repetitive sequences in the *C. sonnerati* genome.

**Identification method**	**Repeat size**	**% of genome**
Trf	43,259,813	4.14
Repeatmasker	155,219,394	14.87
Proteinmask	33,956,519	3.25
De novo	451,894,841	43.28
Total	526,923,565	50.47

Biological classification	Combined TEs	
	Length (bp)	% in genome
DNA	259,138,824	24.82
LINE	143,448,649	13.74
SINE	20,458,386	1.96
LTR	70,157,749	6.72
Other	11,083	0.00
Unknown	107,454,043	10.29
Total TE	493,109,387	47.23

Table S6. Statistics of gene predictions in the *C. sonnerati* genome.

**Gene set**		**Number**	**Average gene length (bp)**	**Average CDS length (bp)**	**Average exon num per gene**	**Average exon length (bp)**	**Average intron length (bp)**
De novo	AUGUSTUS	28,361	18,524.94	1458.67	8.34	174.81	2323.75
	Genscan	32,602	23,337.65	1527.94	8.71	175.41	2828.54
Homolog	*O.niloticus*	46,273	11,823.46	1,146.72	5.76	199.11	2243.32
	E.lanceolatus	43,428	12,386.84	1,166.35	6.13	190.25	2186.91
	L.calcarifer	43,682	12,522.15	1,163.49	6.11	190.38	2222.24
	G.acuticeps	42,462	11,486.95	1,128.77	5.79	195.11	2164.65
	C.lumpus	42,018	12,148.39	1,142.28	6.02	189.61	2,190.58
	E.akaara	41,589	13,846.16	1,206.10	6.57	183.47	2,267.74
	P.georgianus	42,051	12,681.74	1,182.00	5.98	197.59	2,308.20
	P.leopardus	53,773	10170.53	904.85	4.78	189.16	2,449.01
trans.orf/ISOseq		36,352	20,686.78	1,196.28	9.54	274.54	2,116.70
MAKER		26,130	20,599.55	1,585.97	9.58	243.21	2,129.63

Table S7. Statistics of non-coding genes in the *C. sonnerati* genome.

Type		Copy	Average length (bp)	Total length (bp)	% of genome
miRNA		373	84.00	31,331	0.0030
tRNA		2,232	74.25	165,718	0.0159
rRNA	rRNA	169	173.09	29,253	0.0028
	18S	12	885.92	10,631	0.0010
	28S	0	0.00	0	0.0000
	5.8S	6	139.67	838	0.0001
	5S	151	117.77	17,784	0.0017
	8S	0	0.00	0	0.0000
snRNA	snRNA	515	142.20	73,231	0.0070
	CD-box	118	109.08	12,871	0.0012
	HACA-box	70	152.16	10,651	0.0010
	splicing	322	150.64	48,506	0.0046
	scaRNA	5	240.60	1,203	0.0001

Table S8. Statistics of OrthoMCL clusters in *C. sonnerati* and other 15 species.

Species	Total genes	Unclustered genes	Familes	Unique families	Ave. genes per family
C. sonnerati	26,130	1,998	17,125	127	1.41
E. lanceolatus	23,384	124	16,842	12	1.38
P. leopardus	25,248	1,309	17,205	72	1.39
E. akaara	23,923	270	16,674	32	1.42
O. niloticus	29,092	408	17,052	210	1.68
L. calcarifer	25,329	177	17,073	56	1.47
G. acuticeps	23,324	216	15,759	47	1.47
P. georgianus	22,295	221	15,896	28	1.39
C. lumpus	21,013	205	15,973	18	1.30
D. rerio	30,983	790	16,263	446	1.86
S. salar	41,482	2,335	18,087	272	2.16
M. albus	20,203	197	15,634	20	1.28
G. morhua	19,424	751	14,346	19	1.30
O. mykiss	34,363	1,258	16,892	153	1.96
O. latipes	19,669	672	14,383	79	1.32
L. chalumnae	20,932	1,546	14,539	173	1.33

Table S9. Statistics of positive selection genes in *C. sonnerati*.

Table S10. Enrichment of GO terms with expanded gene families in *C. sonnerati*.

Table S11. Enrichment of KEGG pathways with expanded gene families in *C. sonnerati*.

Table S12. The key genes of GO terms with expanded gene families in the sensory system of *C. sonnerati* genome.

Table S13. The tissue-specific expression genes with MAPK signaling pathway in the brain of *C. sonnerati* transcriptome.

Table S14. The tissue-specific expression genes with Calcium signaling pathway in the brain of C. sonnerati transcrip-tome.

Table S15. The tissue-specific expression genes with cAMP signaling pathway in the brain of *C. sonnerati* transcriptome.

Table S16. The tissue-specific expression genes with nervous system in the brain of *C. sonnerati* transcriptome.

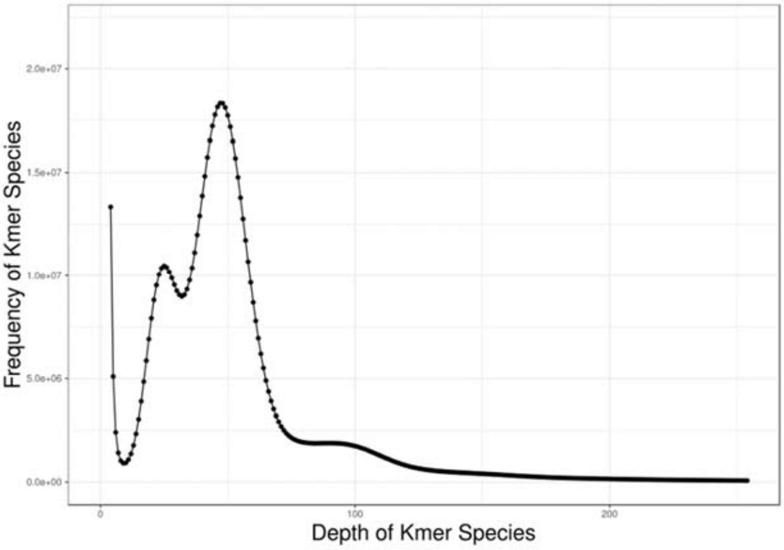

Figure S1. The genome survey of *C. sonnerati* using 17-mer analysis. The highest peak at coverage 47× corresponds t the homozygous peak. The minor peak at coverage 25× corresponds to the heterozygous peak. The minor peak at coverage corresponds to 92× duplications.

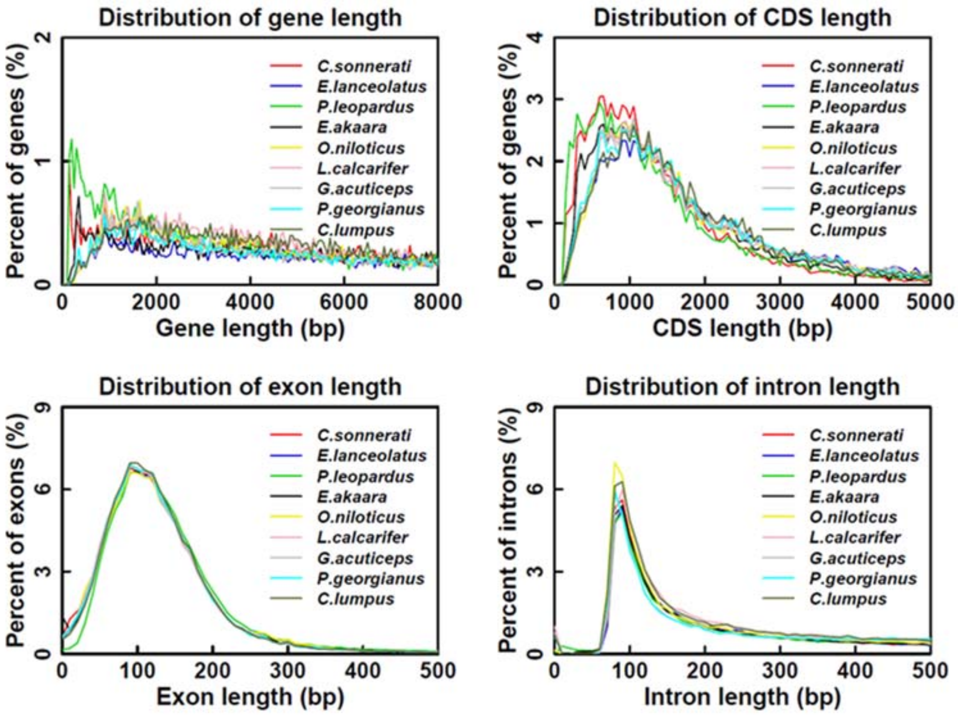

Figure S2. Comparisons of the predicted gene models between *C. sonnerati* genome and other teleost species. (a) gene length. (b) CDS length. (c) exon length. (d) intron length.

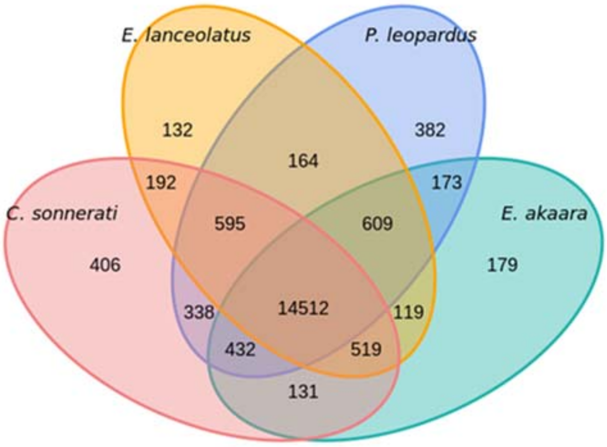

Figure S3. Venn diagram of comparative genomic analyses with the four groupers (*P. leopardus*, *E. akaara*, *E. lanceolatus* and *C. sonnerati*).

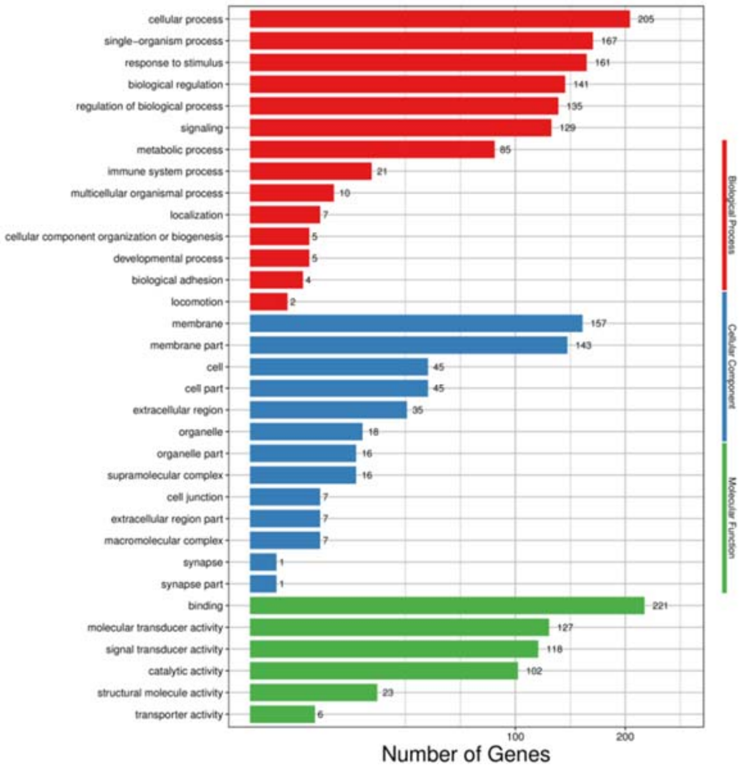

Figure S4. Enrichment of GO terms with expanded gene families in *C. sonnerati* genome ($p<0.05$).

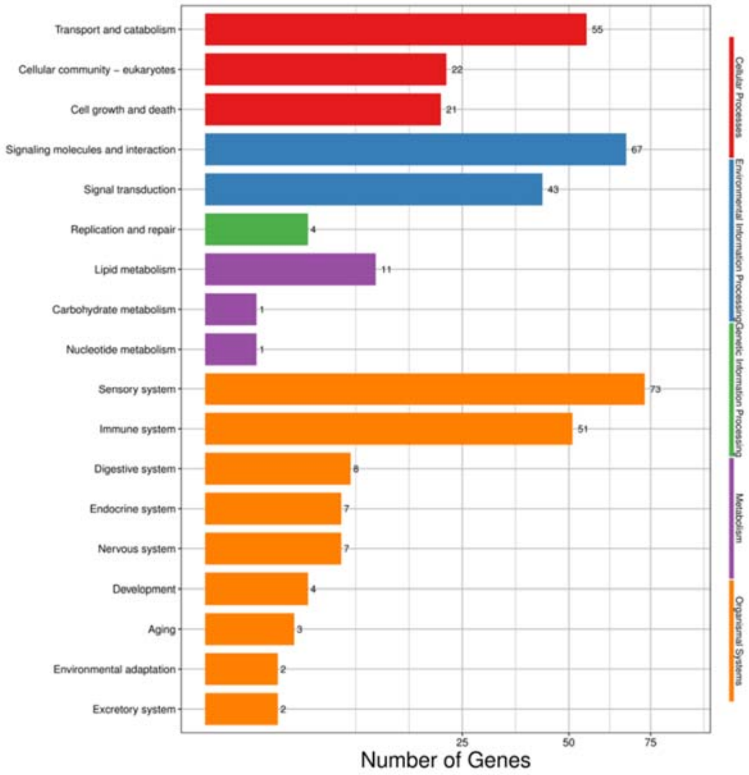

Figure S5. Enrichment of KEGG pathway with expanded gene families in *C. sonnerati* genome ($p<0.05$).

a

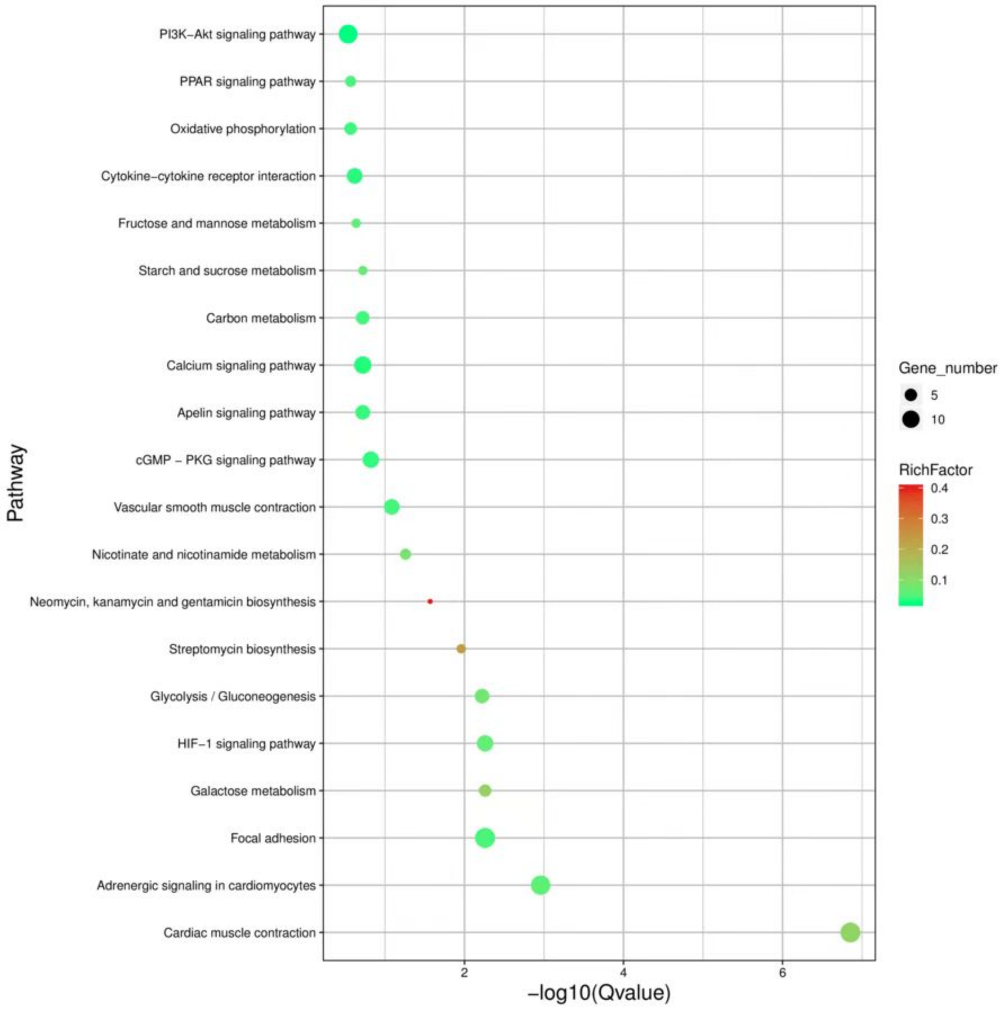
PI3K−Akt signaling pathway
PPAR signaling pathway
Oxidative phosphorylation
Cytokine−cytokine receptor interaction
Fructose and mannose metabolism
Starch and sucrose metabolism
Carbon metabolism
Calcium signaling pathway
Apelin signaling pathway
cGMP − PKG signaling pathway
Vascular smooth muscle contraction
Nicotinate and nicotinamide metabolism
Neomycin, kanamycin and gentamicin biosynthesis
Streptomycin biosynthesis
Glycolysis / Gluconeogenesis
HIF−1 signaling pathway
Galactose metabolism
Focal adhesion
Adrenergic signaling in cardiomyocytes
Cardiac muscle contraction
Pathway
2
4
6
−log10(Qvalue)
Gene_number
5
10
RichFactor
0.4
0.3
0.2
0.1

b

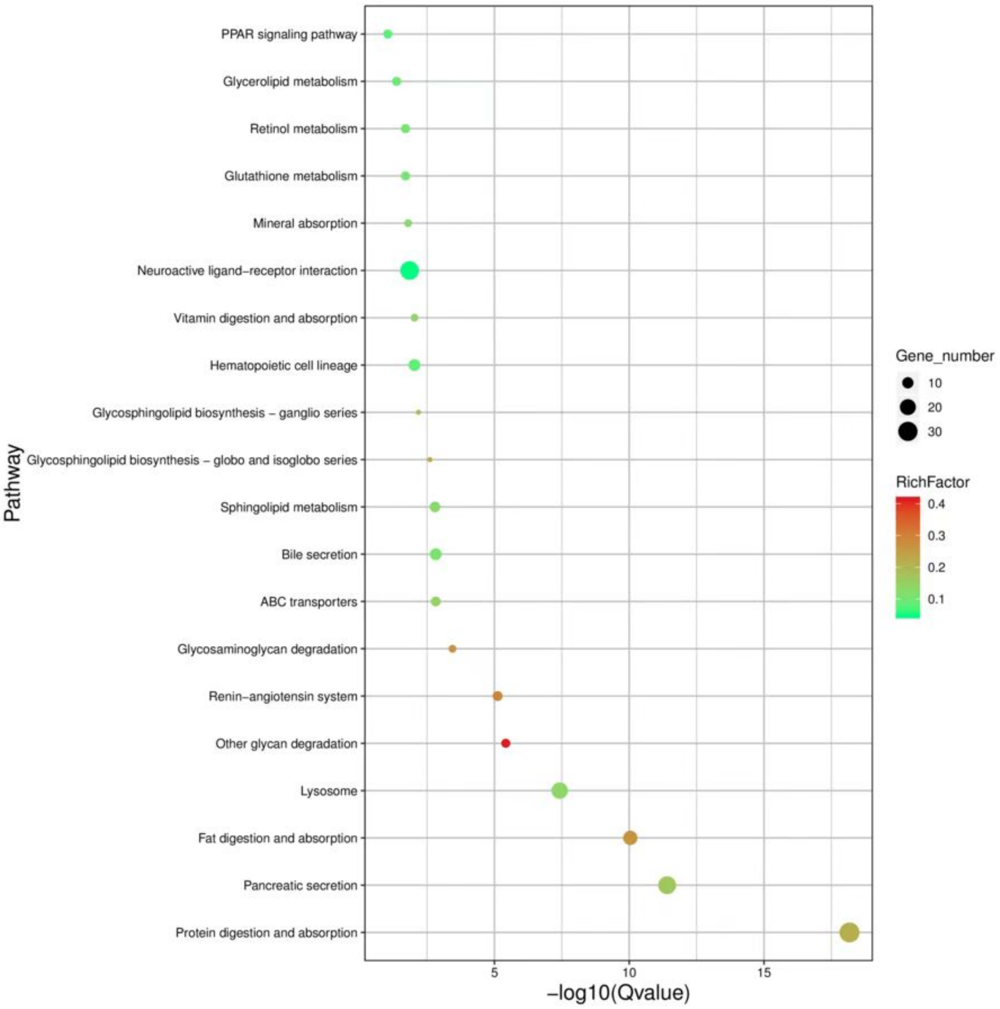
PPAR signaling pathway
Glycerolipid metabolism
Retinol metabolism
Glutathione metabolism
Mineral absorption
Neuroactive ligand–receptor interaction
Vitamin digestion and absorption
Hematopoietic cell lineage
Glycosphingolipid biosynthesis – ganglio series
Glycosphingolipid biosynthesis – globo and isoglobo series
Sphingolipid metabolism
Bile secretion
ABC transporters
Glycosaminoglycan degradation
Renin–angiotensin system
Other glycan degradation
Lysosome
Fat digestion and absorption
Pancreatic secretion
Protein digestion and absorption
Pathway
5
10
15
–log10(Qvalue)
Gene_number
10
20
30
RichFactor
0.4
0.3
0.2
0.1

c

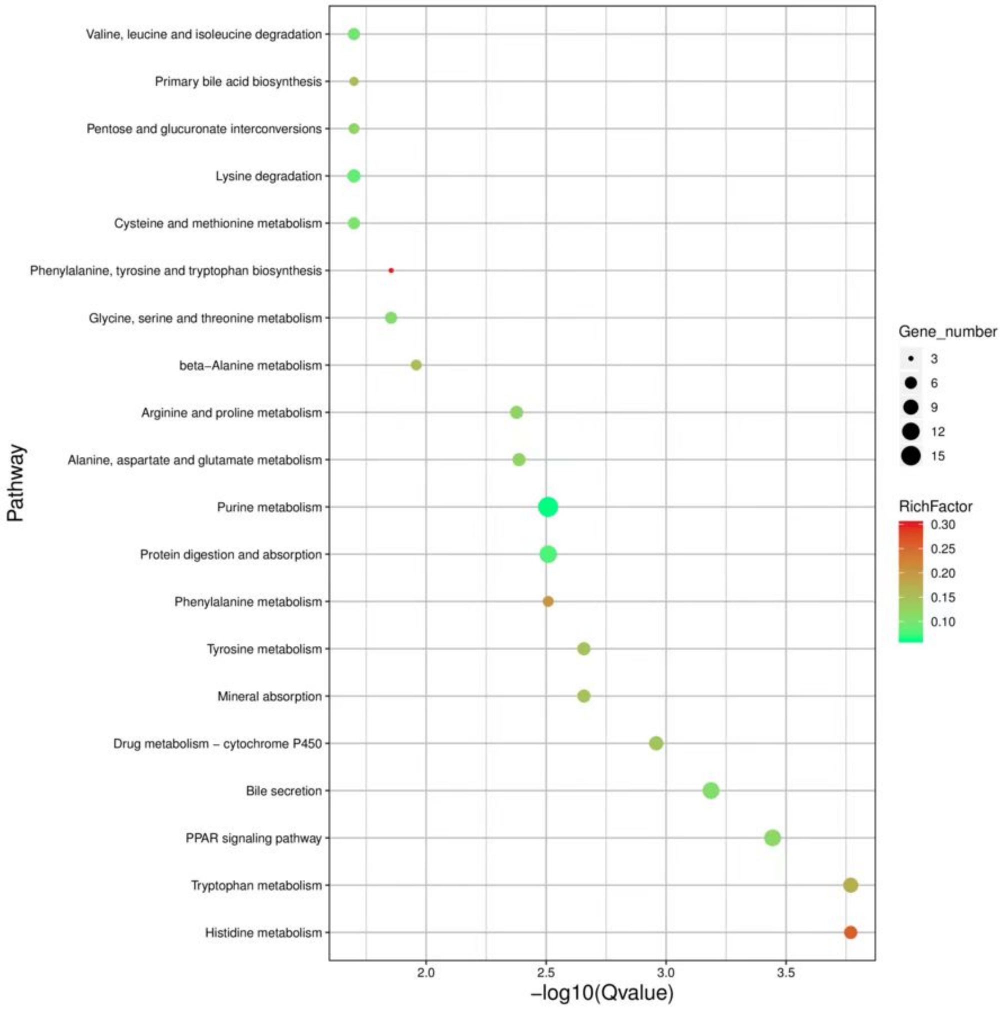

Valine, leucine and isoleucine degradation
Primary bile acid biosynthesis
Pentose and glucuronate interconversions
Lysine degradation
Cysteine and methionine metabolism
Phenylalanine, tyrosine and tryptophan biosynthesis
Glycine, serine and threonine metabolism
beta−Alanine metabolism
Arginine and proline metabolism
Alanine, aspartate and glutamate metabolism
Purine metabolism
Protein digestion and absorption
Phenylalanine metabolism
Tyrosine metabolism
Mineral absorption
Drug metabolism − cytochrome P450
Bile secretion
PPAR signaling pathway
Tryptophan metabolism
Histidine metabolism
Pathway
2.0
2.5
3.0
3.5
−log10(Qvalue)
Gene_number
3
6
9
12
15
RichFactor
0.30
0.25
0.20
0.15
0.10

d

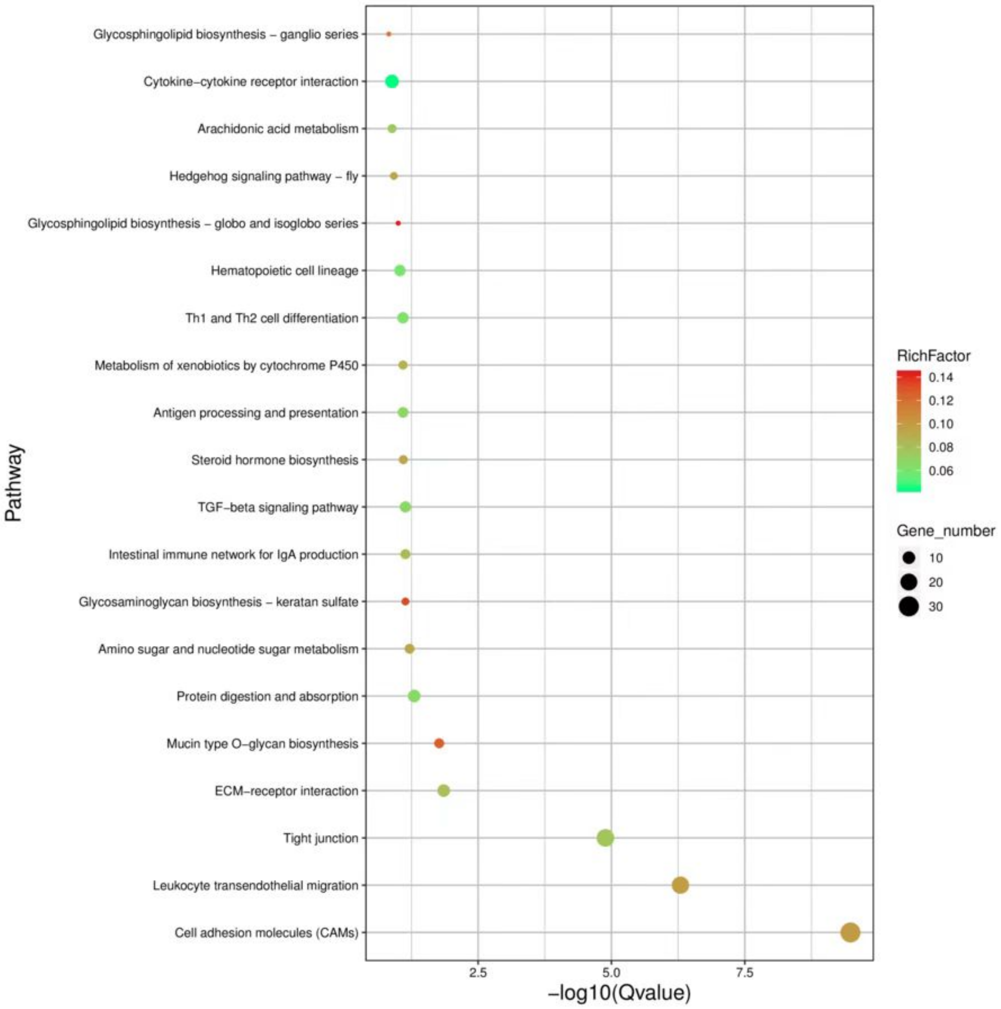
Glycosphingolipid biosynthesis – ganglio series
Cytokine–cytokine receptor interaction
Arachidonic acid metabolism
Hedgehog signaling pathway – fly
Glycosphingolipid biosynthesis – globo and isoglobo series
Hematopoietic cell lineage
Th1 and Th2 cell differentiation
Metabolism of xenobiotics by cytochrome P450
Antigen processing and presentation
Steroid hormone biosynthesis
TGF–beta signaling pathway
Intestinal immune network for IgA production
Glycosaminoglycan biosynthesis – keratan sulfate
Amino sugar and nucleotide sugar metabolism
Protein digestion and absorption
Mucin type O–glycan biosynthesis
ECM–receptor interaction
Tight junction
Leukocyte transendothelial migration
Cell adhesion molecules (CAMs)
Pathway
2.5
5.0
7.5
–log10(Qvalue)
RichFactor
0.14
0.12
0.10
0.08
0.06
Gene_number
10
20
30

e

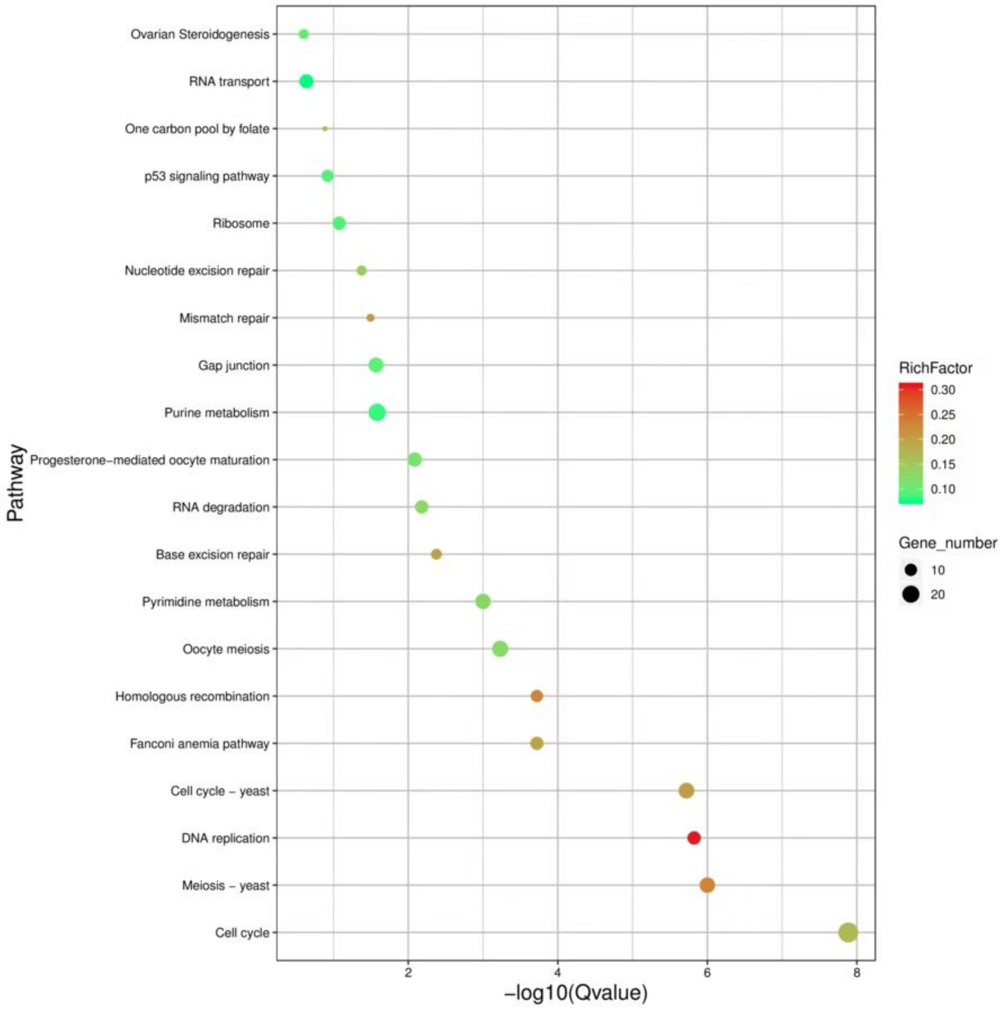
Pathway
Ovarian Steroidogenesis
RNA transport
One carbon pool by folate
p53 signaling pathway
Ribosome
Nucleotide excision repair
Mismatch repair
Gap junction
Purine metabolism
Progesterone−mediated oocyte maturation
RNA degradation
Base excision repair
Pyrimidine metabolism
Oocyte meiosis
Homologous recombination
Fanconi anemia pathway
Cell cycle − yeast
DNA replication
Meiosis − yeast
Cell cycle
2
4
6
8
−log10(Qvalue)
RichFactor
0.30
0.25
0.20
0.15
0.10
Gene_number
10
20

f

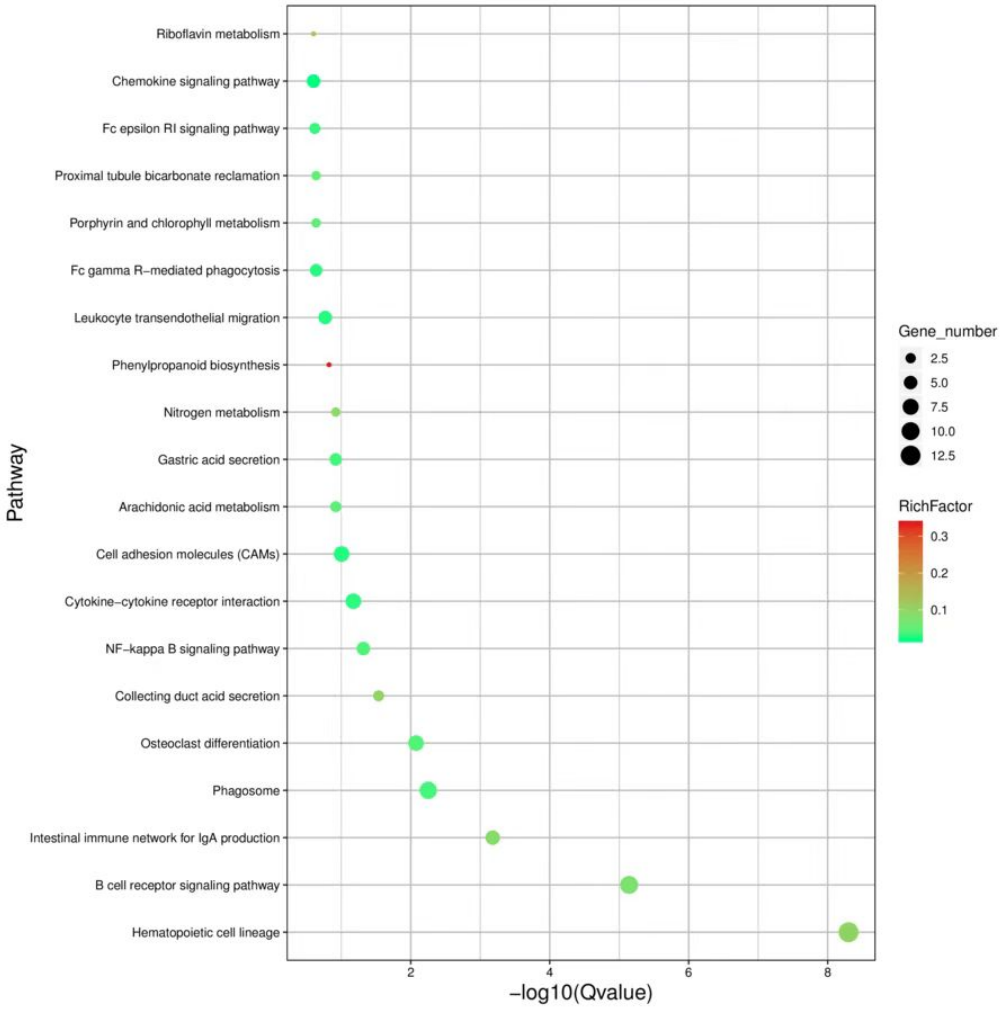
Riboflavin metabolism
Chemokine signaling pathway
Fc epsilon RI signaling pathway
Proximal tubule bicarbonate reclamation
Porphyrin and chlorophyll metabolism
Fc gamma R−mediated phagocytosis
Leukocyte transendothelial migration
Phenylpropanoid biosynthesis
Nitrogen metabolism
Gastric acid secretion
Arachidonic acid metabolism
Cell adhesion molecules (CAMs)
Cytokine−cytokine receptor interaction
NF−kappa B signaling pathway
Collecting duct acid secretion
Osteoclast differentiation
Phagosome
Intestinal immune network for IgA production
B cell receptor signaling pathway
Hematopoietic cell lineage
Pathway
2
4
6
8
−log10(Qvalue)
Gene_number
2.5
5.0
7.5
10.0
12.5
RichFactor
0.3
0.2
0.1

g

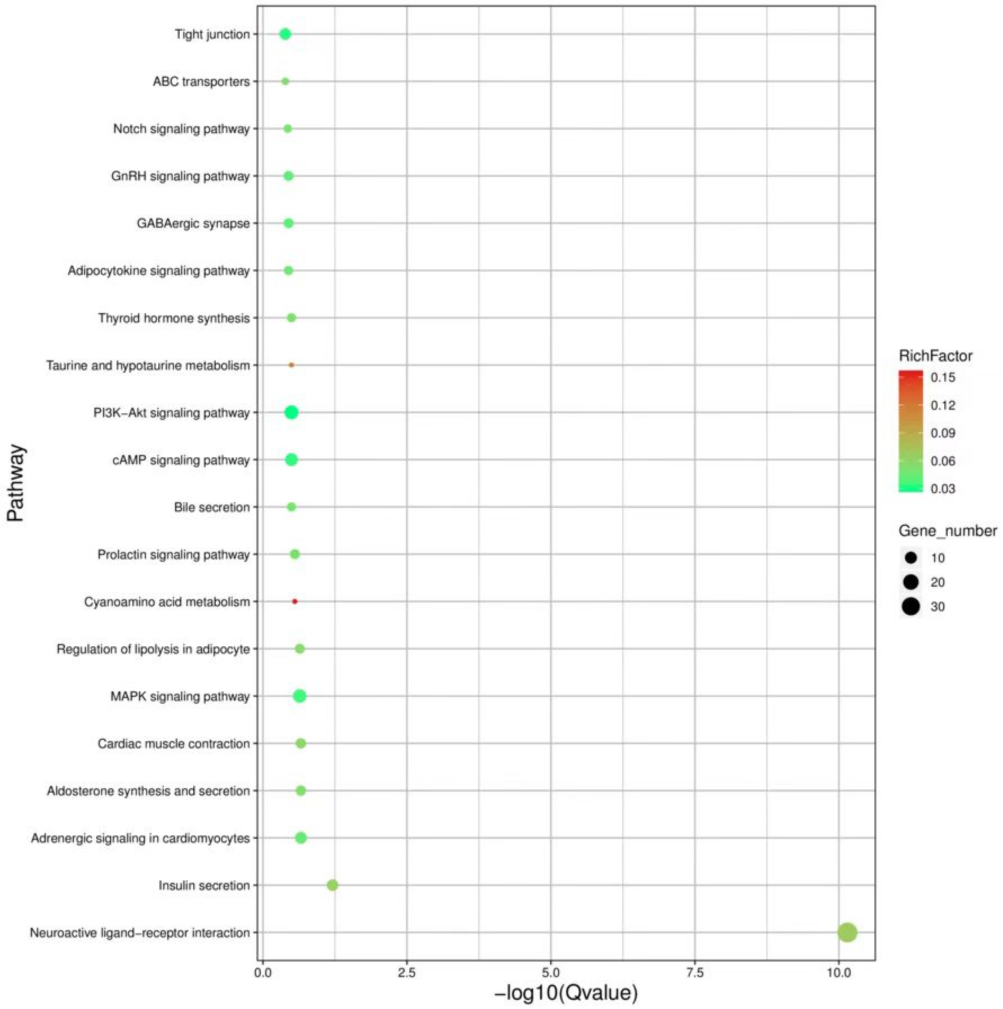
Tight junction
ABC transporters
Notch signaling pathway
GnRH signaling pathway
GABAergic synapse
Adipocytokine signaling pathway
Thyroid hormone synthesis
Taurine and hypotaurine metabolism
PI3K−Akt signaling pathway
cAMP signaling pathway
Bile secretion
Prolactin signaling pathway
Cyanoamino acid metabolism
Regulation of lipolysis in adipocyte
MAPK signaling pathway
Cardiac muscle contraction
Aldosterone synthesis and secretion
Adrenergic signaling in cardiomyocytes
Insulin secretion
Neuroactive ligand−receptor interaction
Pathway
0.0
2.5
5.0
7.5
10.0
−log10(Qvalue)
RichFactor
0.15
0.12
0.09
0.06
0.03
Gene_number
10
20
30

h

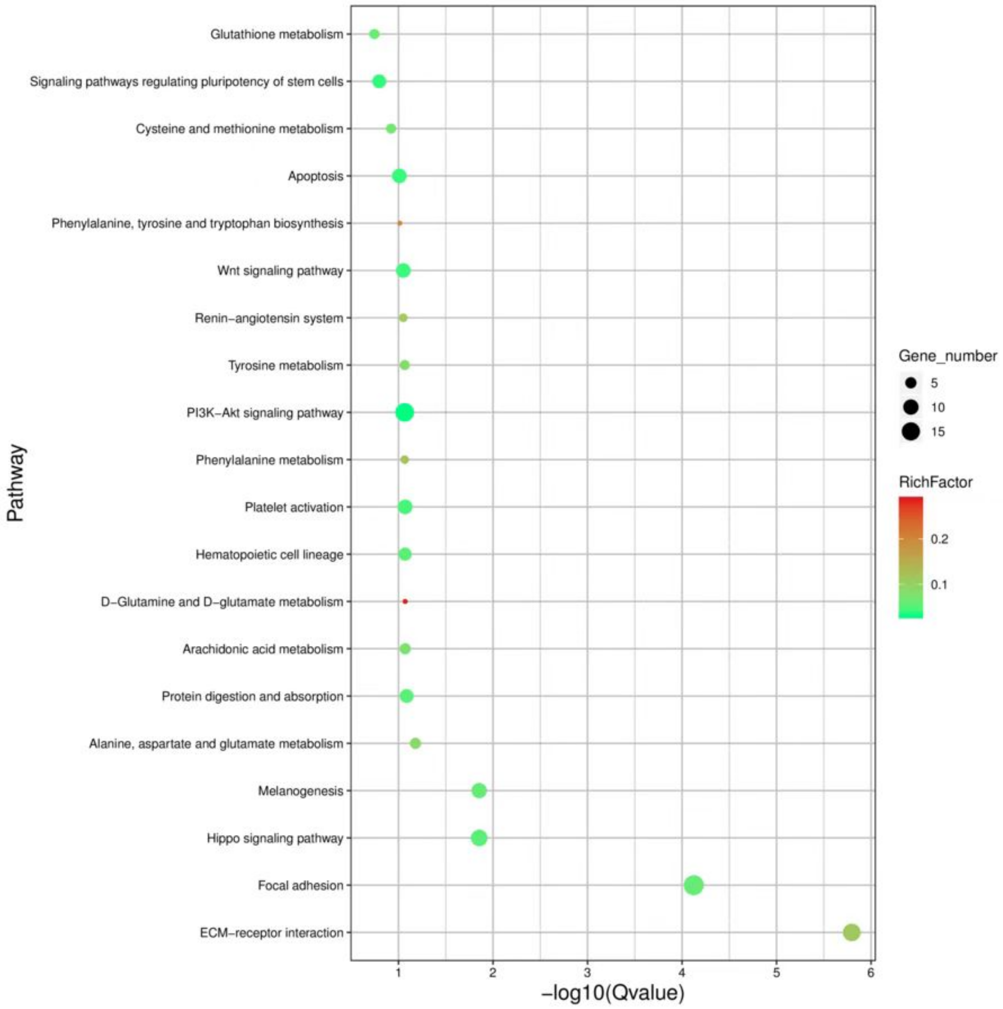

Glutathione metabolism
Signaling pathways regulating pluripotency of stem cells
Cysteine and methionine metabolism
Apoptosis
Phenylalanine, tyrosine and tryptophan biosynthesis
Wnt signaling pathway
Renin−angiotensin system
Tyrosine metabolism
PI3K−Akt signaling pathway
Phenylalanine metabolism
Platelet activation
Hematopoietic cell lineage
D−Glutamine and D−glutamate metabolism
Arachidonic acid metabolism
Protein digestion and absorption
Alanine, aspartate and glutamate metabolism
Melanogenesis
Hippo signaling pathway
Focal adhesion
ECM−receptor interaction
Pathway
1
2
3
4
5
6
−log10(Qvalue)
Gene_number
5
10
15
RichFactor
0.2
0.1

i

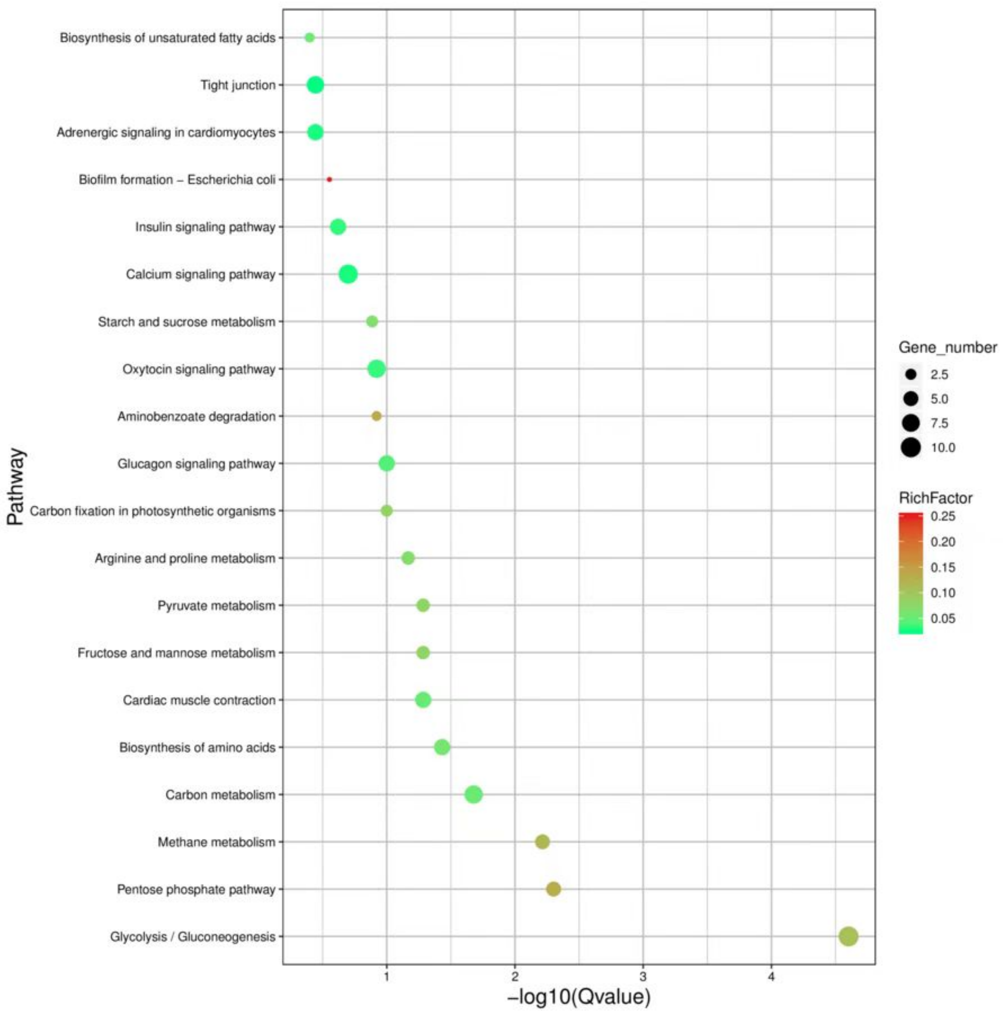
Biosynthesis of unsaturated fatty acids
Tight junction
Adrenergic signaling in cardiomyocytes
Biofilm formation – Escherichia coli
Insulin signaling pathway
Calcium signaling pathway
Starch and sucrose metabolism
Oxytocin signaling pathway
Aminobenzoate degradation
Glucagon signaling pathway
Carbon fixation in photosynthetic organisms
Arginine and proline metabolism
Pyruvate metabolism
Fructose and mannose metabolism
Cardiac muscle contraction
Biosynthesis of amino acids
Carbon metabolism
Methane metabolism
Pentose phosphate pathway
Glycolysis / Gluconeogenesis
Pathway
1
2
3
4
–log10(Qvalue)
Gene_number
2.5
5.0
7.5
10.0
RichFactor
0.25
0.20
0.15
0.10
0.05

j

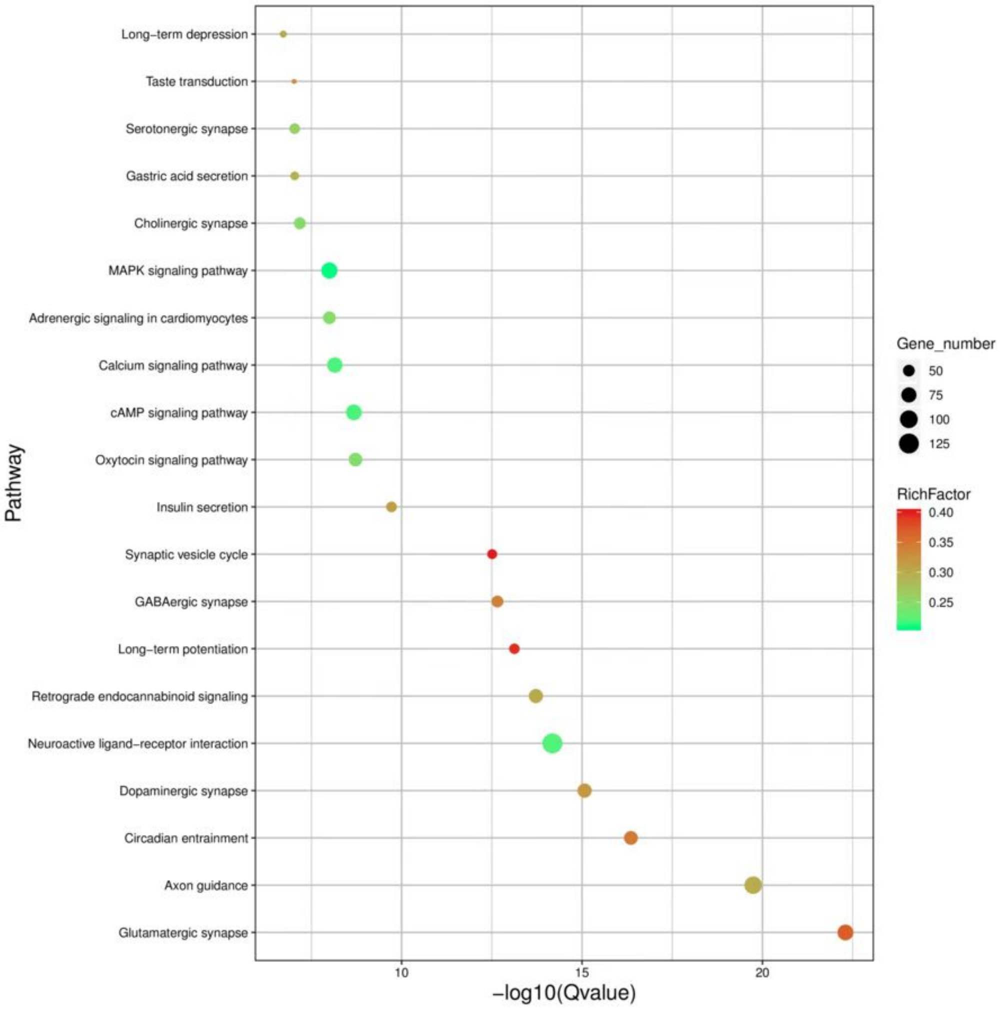
Long−term depression
Taste transduction
Serotonergic synapse
Gastric acid secretion
Cholinergic synapse
MAPK signaling pathway
Adrenergic signaling in cardiomyocytes
Calcium signaling pathway
cAMP signaling pathway
Oxytocin signaling pathway
Insulin secretion
Synaptic vesicle cycle
GABAergic synapse
Long−term potentiation
Retrograde endocannabinoid signaling
Neuroactive ligand−receptor interaction
Dopaminergic synapse
Circadian entrainment
Axon guidance
Glutamatergic synapse
Pathway
10
15
20
−log10(Qvalue)
Gene_number
50
75
100
125
RichFactor
0.40
0.35
0.30
0.25

k

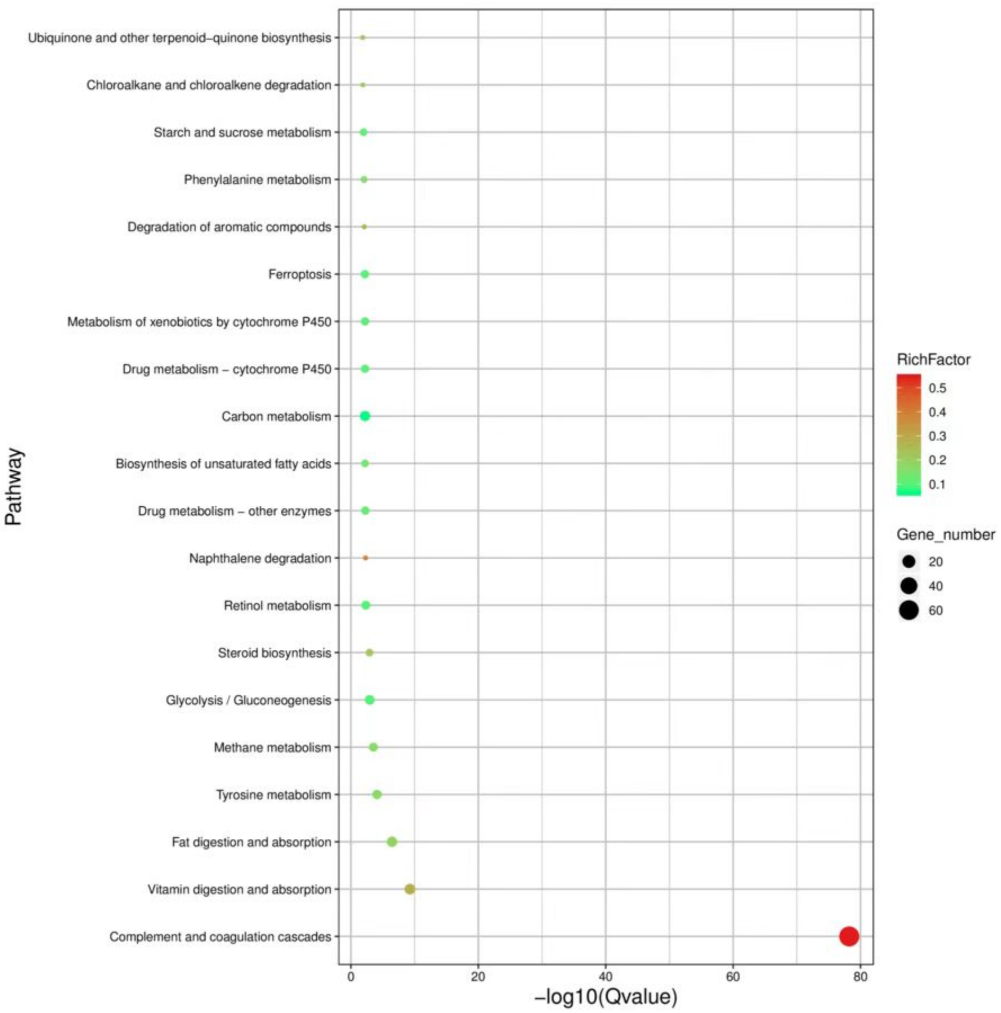

Figure S6. Functional analyses of tissue-specific expression genes using the KEGG database. a) heart DEG enrichment; b) intestine DEG enrichment; c) kidney DEG enrichment; d) gill DEG enrichment; e) gonad DEG enrichment; f) headkidney DEG enrichment; g) pituitary DEG enrichment; h) skin DEG enrichment; i) muscle DEG enrichment; j) brain DEG enrichment; k) liver DEG enrichment.

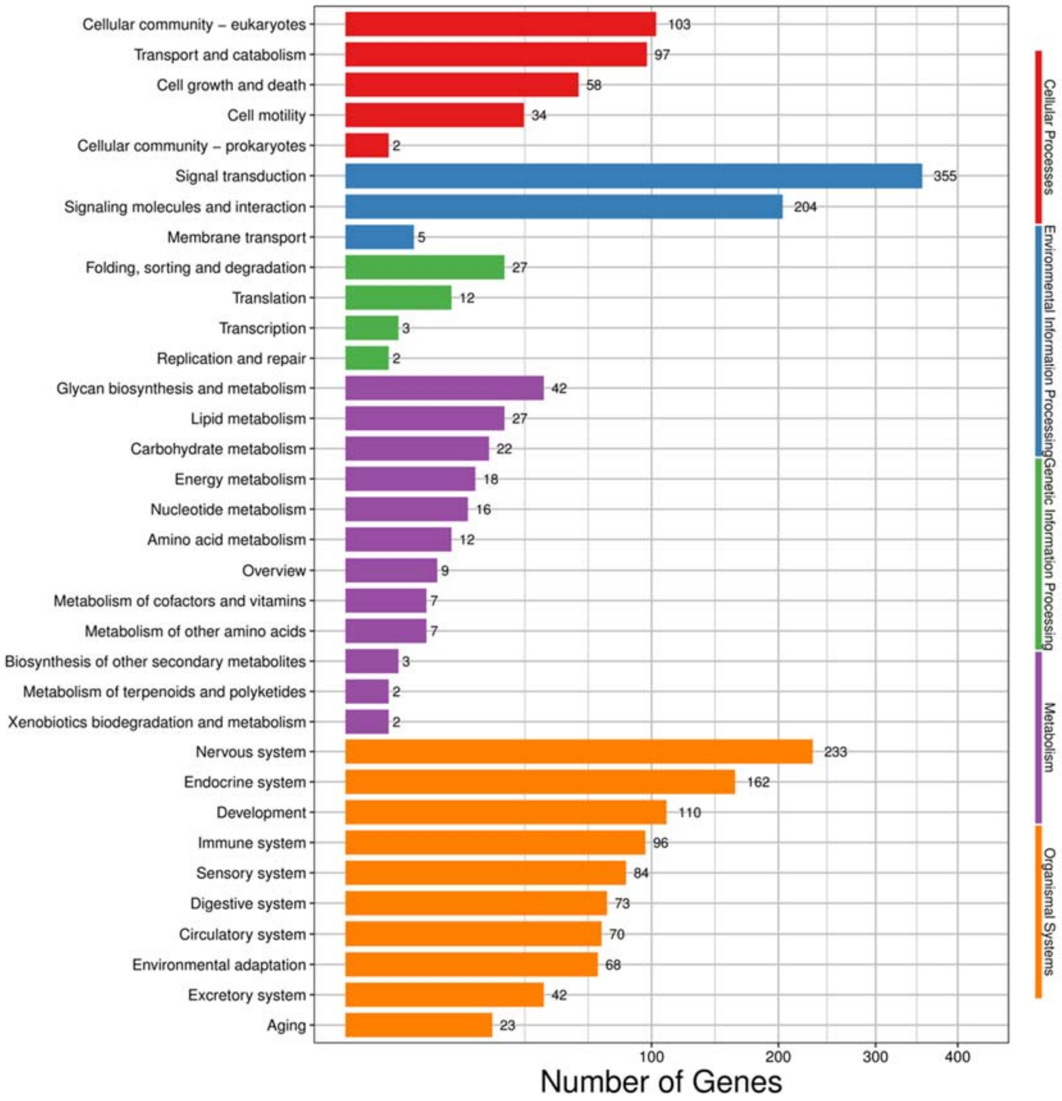

Figure S7. Functional analyses of tissue-specific expression genes in the brain using the GO database.
